# Depression and shortened survival in patients with cancer: a neuroimmune framework for bidirectional pathogenesis and therapeutic interception

**DOI:** 10.3389/fimmu.2026.1927860

**Published:** 2026-09-24

**Authors:** Jiang-Bo Li, Wei Rong, Bin-Hui Xiao, Fan Zhou, Li-Hong Lei, Yu-Wei Liu, Zai-Ping Wang, Sha Zhu

**Affiliations:** 1 Department of Psychosomatic Medicine, Jianyang People’s Hospital, Jianyang, Sichuan, China; 2 Clinical Psychology Department, Wuhu Second People’s Hospital, Wuhu, Anhui, China

**Keywords:** autonomic nervous system, cancer survival, depression, hypothalamic-pituitary-adrenal axis, immunometabolism, systems-level dysregulation, tumor immune microenvironment, β-adrenergic signaling

## Abstract

Although depression is highly prevalent among patients with cancer and is consistently linked to shortened survival, its underlying biological mechanisms remain incompletely understood. While emerging evidence suggests that depression reshapes both systemic and tumor-localized immunity via β-adrenergic and glucocorticoid signaling, the broader integrative pathways remain to be fully defined. To address this gap, this review synthesizes evidence from clinical, epidemiological, and translational studies to present a unifying systems-level framework: depression influences cancer outcomes through the coordinated dysregulation of the autonomic nervous system (ANS), hypothalamic-pituitary-adrenal (HPA) axis, and neuroimmune network. Collectively, this triad fosters a pro-tumorigenic microenvironment. Unlike simpler linear models, this framework emphasizes the coupled, self-reinforcing interactions among these three systems. Specifically, sympathetic overdrive combined with vagal withdrawal amplifies the HPA output, peripheral inflammation sustains central dysregulation, and brain-derived neurotrophic factor (BDNF) dysfunction impairs neuroendocrine feedback. Herein, we critically evaluate the evidence supporting each node of this triad and highlight persistent controversies and evidence gaps, particularly regarding causal directionality and the translational divide between preclinical and human studies. Furthermore, we propose a targeted research agenda introducing three testable endophenotypes (HPA-dominant, inflammation-dominant, and cytotoxicity-dominant) designed to guide future biomarker-stratified immuno-oncology trials. We argue that while restoring autonomic-neuroendocrine-immune homeostasis represents a promising, testable strategy to improve survival, any related therapeutic recommendations remain strictly investigational until validated in large-scale randomized controlled trials with survival endpoints.

## 1 Introduction

Although the past decade has witnessed substantial advances in targeted therapies and immunotherapies, leading to gradual improvements in global cancer survival (1, 2), cancer remains a leading cause of death worldwide. Many patients continue to experience suboptimal outcomes under current treatment paradigms. Even after accounting for established prognostic factors, such as sociodemographic variables, disease stage, treatment modality, nutritional status, and comorbidity burden, substantial inter-individual variability in outcomes remains unexplained (3–6). This residual variance suggests the presence of unrecognized biological drivers that influence cancer progression and patient survival.

To identify and synthesize the available evidence, we searched PubMed, Embase, and PsycINFO from inception to May 2026. The search strategy utilized combinations of the following terms: (“depression”) AND (“cancer” OR “malignancy” OR “carcinoma”) AND (“survival” OR “prognosis” OR “progression”) AND (“autonomic” OR “sympathetic” OR “HPA axis” OR “neuroimmune” OR “BDNF” OR “cytokine” OR “tumor microenvironment”). We prioritized meta-analyses, prospective cohort studies, and mechanistic investigations with human or translational relevance. Although the selected references are not exhaustive, they represent the most biologically informative evidence for each node of the proposed framework.

### 1.1 Models of the link between cancer and depression

In recent years, the comorbidity of depression in patients with cancer has attracted growing attention. Epidemiological surveys report a depression prevalence of 53.21% among patients with cancer in Africa (7) and 33.16% among cancer survivors globally (8). Notably, substantial evidence indicates that comorbid depression is associated with poorer prognoses and significantly shorter survival times (9–13). Meta-analyses consistently demonstrate that depression correlates with reduced survival across diverse malignancies, including prostate cancer after radical prostatectomy (9), acute myeloid leukemia (10), breast cancer (11), glioma, and colorectal cancer (12, 13). The clinical significance of this association is considerable. If the link between depression and poor prognosis reflects a causal or modifiable contribution rather than a mere epiphenomenon, depression represents a highly prevalent, potentially modifiable, and largely underexploited prognostic target (14, 15). Nevertheless, the biological mechanisms by which depression influences cancer outcomes remain incompletely understood and actively debated. Critically, the observed association does not inherently establish causation. Reverse causality, wherein cancer progression or its treatment induces depressive symptoms, remains a plausible alternative explanation that must be rigorously addressed in future studies.

The prevailing neuroendocrine model primarily focuses on hypothalamic-pituitary-adrenal (HPA) axis hyperactivity, positing that cortisol-driven immunosuppression creates a permissive microenvironment for tumor escape (16, 17). Separately, a growing body of work implicates brain-derived neurotrophic factor (BDNF) downregulation as a molecular correlate of both depressive severity and poor cancer prognosis (18, 19). More recently, immunometabolic reprogramming (particularly through interferon regulatory factor 1 (IRF1)) has emerged as a novel node linking chronic stress to impaired CD8^+^T cell infiltration and metabolic dysregulation in the tumor microenvironment (TME) (20). However, several critical questions remain unresolved. First, do these pathways represent independent, parallel events, or do they converge via a shared upstream driver? Recent reviews in this journal have systematically addressed the neuro-immune crosstalk underlying stress-driven tumor progression, emphasizing the coordinated roles of HPA axis dysregulation and sympathetic overdrive in reshaping antitumor immunity (21, 22). Second, what is the fundamental causal direction underlying these associations? Third, if depression truly contributes to cancer progression, why have large-scale trials of antidepressants (including a randomized controlled trial that was prematurely terminated due to a potential survival disadvantage) failed to demonstrate unequivocal survival benefits (23)? Subsequent systematic reviews and meta-analyses in glioma similarly found no significant association between antidepressant use and improved survival (24, 25). These paradoxes suggest that the current pathway-centric view is likely incomplete. Specifically, a key piece of the puzzle, the autonomic nervous system (ANS), a rapidly acting, tumor-infiltrating regulator, has been largely overlooked in prior mechanistic frameworks.

Our preliminary work identified neuropeptide alterations in patients with cancer and animal models with comorbid depression (26–28), suggesting a robust link between neuroendocrine signaling and depressive states. However, these isolated findings lack an integrative framework to explain how neuroendocrine, autonomic, and immune disturbances collectively shape cancer outcomes. This gap is not merely an academic one. Without a unifying model that accounts for the coupled behavior of these networks, clinical trials risk targeting isolated biomarkers while leaving the core pathogenic architecture unchanged. Furthermore, the field currently lacks a clear rationale for the combination of interventions. For example, it remains unclear whether a selective serotonin reuptake inhibitor (SSRI) should be paired with a β-blocker, whether non-pharmacological strategies (e.g., exercise and mindfulness) exert their effects through distinct or overlapping pathways, and how these multimodal interventions might optimally synergize.

In this review, we aim to (a) synthesize existing evidence across clinical, epidemiological, and translational studies to propose a unifying systems-level framework; (b) critically evaluate the evidence for each component of this framework, highlighting controversies and gaps; and (c) outline a testable research agenda to guide future mechanistic and clinical studies. We argue that depression alters cancer outcomes through the coordinated dysregulation of the autonomic, neuroendocrine, and neuroimmune systems, a triad that collectively fosters a pro-tumorigenic milieu. Specifically, we evaluate the prevailing neuroendocrine (HPA-axis), ANS, and neuroimmune models. Our central proposition is a tripartite systems framework that integrates these three components into a single, self-reinforcing network to explain the depression-cancer link and guide future therapeutic strategies. Crucially, we present this framework as a working model that synthesizes existing observational associations to generate testable hypotheses. We discuss the promise and pitfalls of targeting this neuro-immune-endocrine hub with antidepressant and lifestyle interventions and outline a testable agenda to guide future mechanistic and clinical studies. Ultimately, this framework may open a new frontier in oncology: treating the host rather than the tumor alone, by restoring neuro-immune-endocrine homeostasis to concurrently improve mental health and cancer survival.

## 2 A tripartite systems framework linking depression to cancer progression

Before examining individual systems, we present an integrated conceptual model (**Figure 1**). Under chronic cancer-related stress, three interdependent physiological systems are progressively altered: the ANS (predominantly characterized by sympathetic overdrive coupled with vagal withdrawal), HPA axis, and neuroimmune network. Persistent sympathetic activation triggers the release of norepinephrine, which directly stimulates tumor cell proliferation, angiogenesis, and invasion while simultaneously inhibiting cytotoxic T lymphocyte (CTL) and natural killer (NK) cell activity. Concurrently, HPA axis activation produces sustained cortisol elevation, which further suppresses adaptive immunity. These neuroendocrine changes are accompanied by the downregulation of BDNF, which impairs neuroendocrine feedback and potentially disrupts appetite regulation in the hypothalamus. Crucially, these three systems do not operate sequentially; rather, they engage in intensive crosstalk: sympathetic signals amplify HPA output, cortisol sensitizes sympathetic receptors on immune cells, and peripheral inflammation feeds back to the brain to sustain autonomic and endocrine imbalances. The resulting TME, characterized by TH1/TH2 imbalance, reduced CTL/NK function, and elevated interleukin-6(IL-6) tumor necrosis factor-alpha(TNF), and C-reactive protein (CRP), favors tumor progression. In this systems-level view, pathway alterations (e.g., cytokine elevation and BDNF reduction) are not independent causes but rather surface manifestations of a deeper, distributed functional alteration across the three master regulatory systems. In **Figure 1**, solid arrows denote direct stimulatory or inhibitory effects, whereas dashed arrows represent regulatory feedback loops or hypothesized intervention targets. Below, we critically evaluate the evidence for each system and highlight the areas of controversy and evidence gaps. As most of the evidence reviewed below is correlational, we explicitly distinguish between well-supported associations and speculative causal mechanisms.

**Figure 1:**
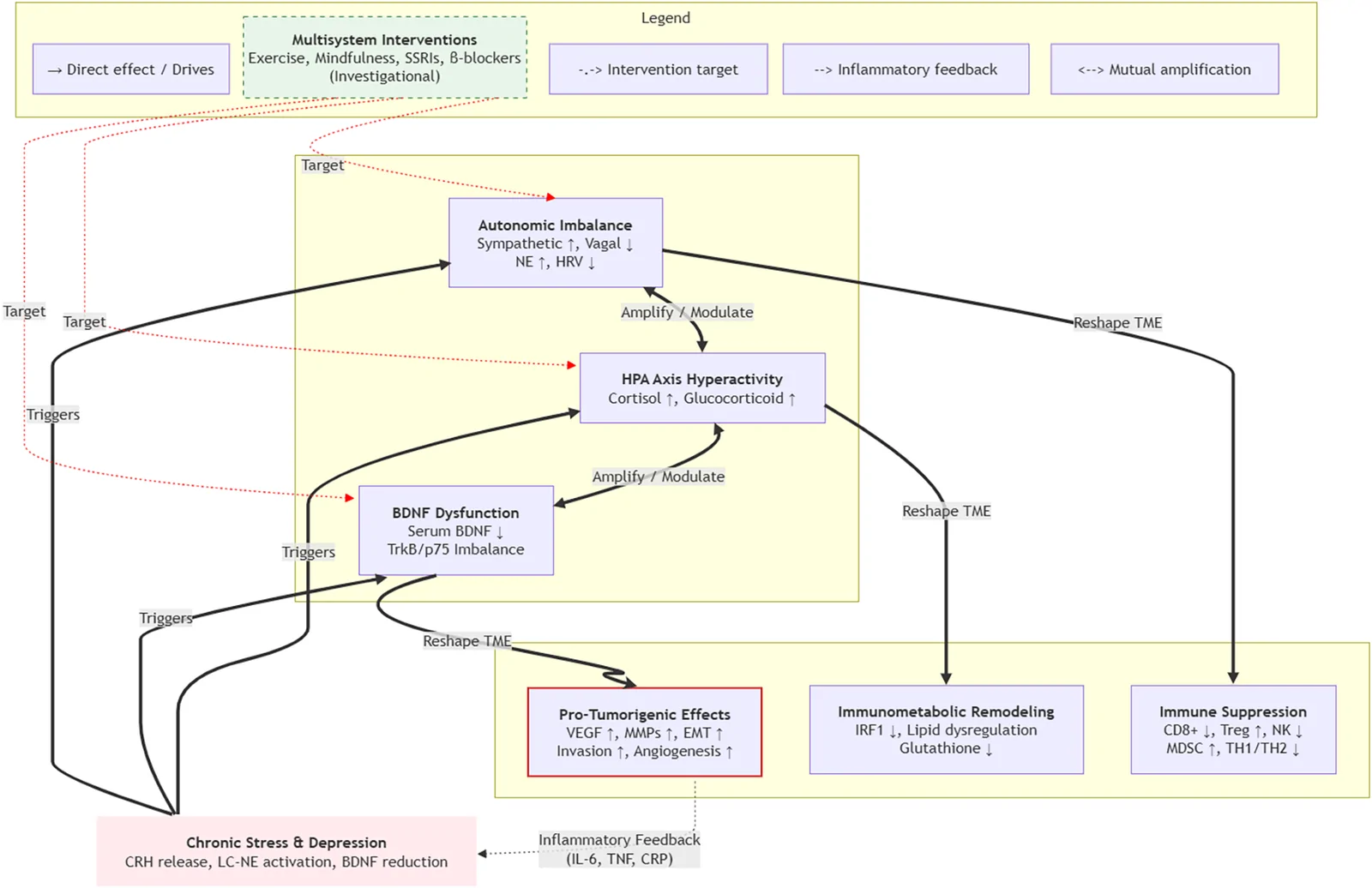
A systems-level neuroimmune network linking depression to cancer progression and cachexia. Chronic stress and depression progressively dysregulate three interdependent master regulatory systems: the autonomic nervous system (sympathetic overdrive coupled with vagal withdrawal), the hypothalamic–pituitary–adrenal (HPA) axis, and the neuroimmune network. Sympathetic activation and HPA axis stimulation elevate cortisol and catecholamines, while reducing brain-derived neurotrophic factor (BDNF). These mediators reshape the tumor microenvironment via three interconnected pathways: (a) autonomic-immune remodeling (decreased CD8^+^/NK cells, increased regulatory T cells/myeloid-derived suppressor cells, and a Th1/Th2 shift), (b) immunometabolic remodeling (decreased IRF1 expression), and (c) BDNF dysfunction. The gut microbiome has emerged as an additional node linking depression to systemic inflammation and immunotherapy resistance, although direct evidence in this context remains limited. Crucially, these systems do not operate in isolation; sympathetic signals amplify HPA output, and peripheral inflammation feeds back to sustain central dysregulation. Collectively, these factors drive cancer progression and cachexia. In turn, elevated inflammatory cytokines (IL-6 and TNF), which feed back to the brain to perpetuate autonomic and HPA hyperactivity (red curved arrow), forming a self–reinforcing inflammation–depression–cachexia loop. Multisystem interventions (exercise, mindfulness, SSRIs, and β-blockers) are indicated on the left as investigational strategies targeting all three systems (green dashed arrows). Solid arrows indicate direct stimulatory effects; dashed arrows indicate regulatory/feedback interactions or intervention targets. This integrated systems–level model proposes that depression is not merely a comorbid symptom but a biological driver of cancer progression through the coupled dysregulation of autonomic, neuroendocrine, and neuroimmune networks.

### 2.1 Stress-related autonomic and neuroendocrine dysregulation: from association to mechanism

Cancer-induced inflammation and the psychological burden of diagnosis/treatment activate both the sympathetic nervous system and the HPA axis, increasing norepinephrine and cortisol release (16). A substantial body of correlative evidence links high cortisol levels to hippocampal serotonin system dysfunction, reduced neurotrophic factors, and depressive symptomatology (29, 30). However, most human studies are cross-sectional, leaving the direction of causality uncertain. Conversely, it is equally important to recognize that cancer and its treatment can cause depression (29, 31). The systemic inflammation associated with tumor growth, as well as the side effects of cancer therapies (e.g., fatigue, pain, neurotoxicity), can directly induce sickness behavior and depressive symptoms through neuroimmune activation (32). This is not merely a psychological reaction; pro-inflammatory cytokines released into the periphery can access the brain and induce a syndrome of depression and fatigue (32). Therefore, the relationship between depression and cancer is likely a bidirectional feedback loop, where disease and mood disorders can perpetuate each other (14). Animal models provide stronger causal evidence: Chen et al. (33) demonstrated that stress-induced decreases in 5-HT, increases in IL-6, IL-17A, and norepinephrine, and the downregulation of histidine decarboxylase promote ovarian cancer progression via the IL-6/STAT3/S100A9 pathway in an ovarian cancer mouse model.

A critical unanswered question is whether stress-induced autonomic and neuroendocrine changes directly accelerate tumor growth in humans or whether they primarily impair treatment adherence and health behaviors. In one study, patients with breast cancer had higher perceived stress scores than those with benign breast disease and healthy controls, and their odds of developing depression were 3.57 times higher than those of healthy controls (34). A comprehensive systematic review and meta-analysis of 54 studies (n=5,017) demonstrated that elevated peripheral blood IL-6 (standardized mean difference (SMD) = 0.59; 95% confidence interval (CI), 0.35–0.82), TNF (SMD = 0.73; 95% CI, 0.35–1.11), and CRP (SMD = 0.57; 95% CI, 0.27–0.87) levels are significantly associated with depressive symptom severity across diverse cancer types (32). However, these inflammatory markers are also elevated in cancer, irrespective of depression, and the cross-sectional design of most included studies precludes causal inference. The most parsimonious interpretation is a positive feedback loop: stress → sympathetic and HPA activation → inflammatory cytokines → depressive symptoms → more stress (35); however, this remains a hypothesis requiring prospective testing.

Importantly, the ANS provides a rapid, parallel pathway that may amplify and sustain HPA-driven effects. In patients with cancer and comorbid depression, elevated sympathetic tone is independently associated with increased intratumoral norepinephrine levels (36, 37), enhanced vascular endothelial growth factor (VEGF) expression (38, 39), and reduced intratumoral CD8+ T cell infiltration (40).

Beyond sympathetic overdrive, reduced vagal (parasympathetic) activity constitutes the other half of the autonomic imbalance. The vagus nerve exerts anti-inflammatory effects via the cholinergic anti-inflammatory pathway (α7 nicotinic acetylcholine receptor-dependent inhibition of macrophage TNF, IL-6, and HMGB1 release). In chronic stress and depression, heart rate variability (HRV), a non-invasive index of vagal tone, is consistently reduced, and low HRV has been independently associated with increased all-cause mortality in diverse patient populations. Specifically, the high-frequency component of HRV reflects the parasympathetic (vagal) tone and has emerged as a prognostic indicator in cancer populations. Low HRV, indicative of sympathetic predominance, has been independently associated with elevated circulating IL-6 and CRP levels (41), suggesting a direct link between autonomic imbalance and systemic inflammation. Importantly, HRV is modifiable; mindfulness-based stress reduction and aerobic exercise have consistently increased HRV in cancer survivors, accompanied by reductions in pro-inflammatory cytokines (42). These observations support the inclusion of HRV as an exploratory biomarker in future neuroimmune trials, as it offers a non-invasive, cost-effective, and dynamically responsive readout of autonomic function that could guide the intervention intensity and timing. Thus, the autonomic contribution to tumor progression should be conceptualized as sympathetic-to-vagal imbalance rather than sympathetic overdrive alone. This distinction has therapeutic implications: strategies aimed at enhancing vagal tone (e.g., HRV biofeedback and vagus nerve stimulation) may complement β-blockade and yield synergistic effects, although this remains entirely investigational in the oncology setting. This autonomic imbalance (sympathetic excess coupled with vagal withdrawal) constitutes a previously underappreciated but highly druggable candidate driver of cancer progression. Notably, non-pharmacological interventions, such as exercise and mindfulness, have been shown to restore HRV and attenuate sympathetic tone, suggesting that autonomic function is modifiable through multiple pathways. However, we emphasize that the evidence for ANS as an independent prognostic factor in human cancer comes predominantly from preclinical models and retrospective cohorts; prospective interventional studies with ANS-targeted stratification are lacking.

### 2.2 The inflammation-depression-cachexia vicious cycle: a testable hypothesis

Beyond its direct effects on tumor growth and immune evasion, this neuroendocrine dysregulation may indirectly accelerate cancer progression by promoting cachexia, a multifactorial wasting syndrome that affects patients with variable cancer types and dramatically worsens prognosis (43). We propose that depression may accelerate and exacerbate cachexia through two mechanisms: (a) depression-associated anorexia and fatigue may synergize with cachexia-induced metabolic dysregulation (44) and (b) shared autonomic and neuroendocrine drivers (elevated norepinephrine, cortisol, and catecholamines) and inflammatory mediators (IL-6 and TNF) contribute to both conditions (45, 46). This clinical relevance is reinforced by a recent systematic review and meta-analysis of human breast cancer studies, which reported that elevated IL-6 levels were consistently associated with poor overall survival (pooled hazard ratio (HR) = 2.25, 95% CI 1.83–2.76), alongside immunosuppressive cytokines such as IL-10 and TGF-β that facilitate tumor immune evasion (47). This bidirectional relationship is particularly well-illustrated in gastric cancer, where depression may serve both as a predisposing factor for disease onset and as a consequence of cancer-related metabolic deterioration (31).

A novel link involves BDNF. Depression-associated BDNF downregulation (18, 19) can disrupt hypothalamic appetite regulation, potentially facilitating the transition from pre-cachexia to refractory cachexia. However, direct evidence of BDNF-mediated effects on cancer cachexia in humans is lacking; thus, we identified this as a priority for future research. It is equally plausible that cachexia-induced systemic inflammation drives BDNF changes and depressive symptoms via reverse causality, an alternative that we explicitly acknowledge.

The bidirectional links among inflammation, depression, and cachexia are biologically plausible; however, clinical efforts to break this cycle have proven to be challenging. A recent double-blind placebo-controlled randomized trial found that low-dose mirtazapine (15 mg nightly for 8 weeks) did not improve appetite or weight in patients with advanced cancer and established cachexia, despite its known antidepressant and orexigenic properties (48). Importantly, this trial excluded patients with significant depressive symptoms (depression score ≤3 on a 0–6 scale), which may have inadvertently removed the subgroup in which an antidepressant might exert differential effects. This negative result suggests that biological mechanisms alone are insufficient to guide clinical practice; patient selection (pre-cachexia vs. refractory cachexia), depression comorbidity status, and timing of intervention may be as critical as drug choice.

We hypothesized that intervening in depression during the pre-cachexia stage rather than waiting until weight loss is established could delay or prevent cachexia progression. This hypothesis is testable in prospective cohorts that serially assess depressive symptoms, inflammatory markers, BDNF levels, autonomic function (e.g., HRV), and body composition.

### 2.3 BDNF: a bidirectional molecular switch

BDNF is traditionally known for its neurotrophic functions (49). However, it also plays complex, context-dependent roles in cancer. We critically evaluated the evidence for seemingly contradictory findings. BDNF is overexpressed in a wide range of malignancies, including lung, breast, ovarian, colorectal, gastric, pancreatic, and glioblastoma, where it promotes proliferation, migration, invasion, and angiogenesis via tropomyosin receptor kinase B (TrkB) signaling (50). In contrast, other studies have reported BDNF downregulation in gastric and colorectal cancers (49, 51).

We suggest that this paradox may be resolved by considering two factors: (a) differential receptor expression (TrkB signaling promotes survival/proliferation, whereas p75 neurotrophin receptor (p75NTR) can induce apoptosis) and (b) compartmental context (systemic BDNF levels may differ from intratumoral BDNF). As p75NTR and TrkB are the primary receptors that mediate BDNF signaling, their relative expression levels determine whether BDNF promotes survival or apoptosis. This leads to a testable prediction: the net effect of BDNF on cancer progression depends on the tumor type’s TrkB/p75NTR ratio and on whether BDNF is measured in serum, plasma, or tumor tissue. Future studies should explicitly report these parameters.

An important question remains unanswered: is the decline in serum BDNF observed in patients with cancer and comorbid depression functionally linked to tumor tissue BDNF signaling? Circulating BDNF is primarily derived from platelets and neurons, whereas tumor tissue BDNF may be produced locally by cancer cells or stromal components. As these two pools may operate independently through distinct receptor systems, the simplified linear chain of “depression → reduced serum BDNF → tumor progression” remains to be functionally established, and its position within the systems framework requires further experimental verification. Low BDNF levels are associated with poor prognosis in multiple myeloma (52) and small cell lung cancer (19) in the context of comorbid depression. In lung cancer-susceptible mice, a combination of chronic unpredictable mild stress and cigarette smoke exposure promotes lung cancer development and induces more severe depression-like behaviors and pathological changes (53). Abnormal glucocorticoid secretion, inflammatory responses, and BDNF dysfunction may serve as common biological mediators between depression and lung tumorigenesis. These clinical observations of low BDNF in patients with cancer and comorbid depression raise the question of whether BDNF restoration could be protective. Animal studies provide preliminary mechanistic support: environmental enrichment promotes IL-15 and BDNF synthesis, and intracranial BDNF injection reduces glioma migration via truncated TrkB.T1 receptors (54). Importantly, intervention studies have demonstrated that BDNF is modifiable: 16 weeks of yoga/meditation significantly increased serum BDNF in cancer survivors (55), and a meta-analysis showed that 6 weeks of antidepressant treatment significantly elevated serum BDNF in depressed patients (18). Thus, BDNF dysfunction may serve as a key molecular link between depression and cancer progression and a potential therapeutic target. Beyond its neurotrophic functions, BDNF also modulates microglial activation and neuroinflammation, which may indirectly shape systemic antitumor immunity. However, the link between BDNF-mediated neuroimmune crosstalk and peripheral TME remodeling remains speculative and requires dedicated investigation. Ultimately, as the contradictory findings regarding the role of BDNF in cancer may stem from the independent regulation of its peripheral and intratumoral pools, a core translational research gap is the lack of studies using paired clinical specimens to simultaneously measure serum and intratumoral BDNF. Such studies are essential to determine the functional relationship between these two compartments.

### 2.4 Immune and immunometabolic remodeling: from autonomic and neuroendocrine signals to the TME

Immune and immunometabolic remodeling is the most biologically plausible and mechanistically detailed component of the three systems. The conceptual chain is as follows: chronic stress → sympathetic and HPA activation → elevated norepinephrine and cortisol/catecholamines → direct effects on immune cells → immunosuppressive TME (17). However, most human evidence remains inconclusive. A prospective cohort study showed that a high Advanced Lung Cancer Inflammation Index (reflecting better nutritional/inflammatory status) was associated with reduced mortality only in cancer survivors without depression (56), suggesting that depression modifies the prognostic value of systemic inflammation.

The reciprocal interaction between the nervous and immune systems in the TME has been the focus of recent comprehensive reviews (22), with particular attention to breast cancer models, where β-adrenergic signaling drives immune escape through multiple convergent mechanisms (57). Mechanistically, chronic stress-induced norepinephrine activates β2-adrenergic receptors (β2-AR) on CD8^+^T cells, leading to PKA-dependent phosphorylation of CREB and subsequent downregulation of T-bet and perforin expression. This signaling cascade reduces the cytotoxic capacity of tumor-infiltrating lymphocytes (TILs) without necessarily depleting their numbers (58). Concurrently, β2-AR signaling on tumor-associated macrophages (TAMs) promotes M2 polarization, increasing IL-10 and arginase-1 production while suppressing IL-12 (59). These dual effects (impaired CTL function and enhanced immunosuppressive macrophage activity) collectively reshape the tumor immune microenvironment (TIME) toward a pro-tumorigenic state.

Strong mechanistic evidence comes from Lutgendorf et al. (40), who showed that depression in patients with ovarian cancer is associated with a reduced TH1 (IFNγ) to TH2 (IL-4) ratio in CD4+ cells and a reduced type 1 cytotoxic T cell (TC1, IFNγ) to type 2 cytotoxic T cell (TC2, IL-4) ratio in CD8+ cells. These findings directly link depression to impaired adaptive immunity in the TME. A systematic review indicated that patients with depression exhibit alterations in immune cell counts, including increased leukocytes, neutrophils, and monocytes and a higher CD4/CD8 ratio (60, 61). The immune-related gene CD3G is upregulated in various cancers and is associated with immune infiltration and oncogenic pathways (62). Most patients with head and neck cancer exhibit T cell dysfunction (reduced CD3ζ expression), which is associated with poor prognosis (63).

Perhaps the most exciting recent advance is the identification of interferon regulatory factor 1 (IRF1) as a molecular node linking chronic stress to immunometabolic reprogramming. The upstream-to-downstream cascade begins with the binding of stress hormones to their receptors on T cells and tumor cells (17): β2-adrenergic receptor (β2-AR) activation by norepinephrine stimulates the cAMP/PKA/CREB pathway (36, 58), while glucocorticoid receptor activation by cortisol leads to transcriptional repression (30, 64). Both of these signaling pathways converge to downregulate the transcription of the IRF1 gene (20). Zheng et al. (20) demonstrated that chronic stress downregulates IRF1 in ovarian cancer models, impairing CD8^+^T cell infiltration and disrupting lipid and glutathione metabolism. High IRF1 expression predicts longer ovarian cancer survival; thus, depression may be associated with ovarian cancer progression by remodeling the immunometabolic microenvironment (20). The discovery of IRF1 as a stress-sensitive immunometabolic node has immediate translational implications. IRF1 expression is not only a prognostic biomarker but also a potential predictive marker for ICI responsiveness, given its role in CD8^+^T cell trafficking and interferon-γ signaling. If depression downregulates IRF1 through chronic glucocorticoid exposure, then patients with comorbid depression and low IRF1 expression may represent a subgroup that derives minimal benefit from ICI monotherapy (20). This generates a testable hypothesis: combining IRF1-modulating agents (e.g., histone deacetylase inhibitors) with ICIs could overcome stress-induced immunotherapy resistance, although this remains entirely preclinical.

Extending this logic, sympathetic overdrive directly remodels the TME by (a) inducing β2-adrenergic receptor signaling on tumor cells to upregulate pro-metastatic genes (e.g., MMPs, VEGF); (b) suppressing CTL and NK cell cytotoxicity; and (c) promoting the accumulation of immunosuppressive cells (Tregs, MDSCs). Preclinical models have shown that chemical or surgical sympathectomy partially reverses these effects, while β-blocker administration reduces tumor growth and synergizes with immune checkpoint inhibitors. Therefore, capturing the full picture of depression-associated immunometabolic remodeling requires integrating the ANS as a core node.

A further, still-emerging node is the gut microbiome, which has been linked to both depressive disorders and cancer immunotherapy responsiveness; however, direct evidence connecting the microbiome to depression-driven inflammation or immunotherapy resistance remains limited (65, 66).

A provocative but underexplored hypothesis is that depression may serve as a predictive biomarker for immunotherapy resistance. Depression-associated inflammatory phenotypes (elevated IL-6 and TNF levels) are known to correlate with primary resistance to ICIs. Conversely, selective serotonin reuptake inhibitors (SSRIs), such as fluoxetine, have immunomodulatory properties that may sensitize tumors to ICIs. β-blockers, which antagonize sympathetic effects on the TME, represent another promising adjunct. This raises the possibility that patients with cancer and comorbid depression constitute a “high-risk but high-benefit” subgroup: without depression treatment, they may respond poorly to ICIs; with concurrent depression treatment (SSRI ± β-blocker), they could achieve better-than-expected responses. This hypothesis requires testing in phase II/III trials that stratify by depression severity and randomize to ICI ± multisystem intervention, with careful safety monitoring for potential immune-related adverse events.

### 2.5 Intervention studies: proof-of-principle for reversibility

If immunometabolic remodeling associated with depression is reversible, interventions that alleviate depressive symptoms should attenuate these immune abnormalities. The following intervention studies provide proof-of-principle evidence for this reversibility. Non-pharmacological interventions (e.g., exercise and mindfulness) reduce IL-6, TNF, and CRP levels, increase NK cell activity, attenuate sympathetic tone, restore heart rate variability, and improve quality of life (67–69). Red ginseng powder may modulate CD3 and CD4 activity during postoperative chemotherapy in patients with advanced gastric cancer, thereby improving survival rates (69). These effects are modest but consistent. Preclinical studies have shown that fluoxetine reduces triple-negative breast cancer viability, inhibits tumor growth, and enhances CTL activity by suppressing Src/JAK/NF-κB signaling (70). Major depressive episodes are associated with increased mortality in all cancer types, and the use of antidepressants may improve the quality of life and potentially increase overall survival (48). However, we emphasize a critical caveat: almost all evidence for the direct antitumor effects of antidepressants comes from preclinical models, and their long-term safety and efficacy in patients with cancer remain unproven. The potential role of β-blockers in adjunctive cancer therapy is primarily supported by retrospective and preclinical studies. Large-scale randomized controlled trials (RCTs) with survival endpoints are necessary before clinical recommendations can be made.

However, most of these interventions have demonstrated biomarker modulation in peripheral blood rather than a direct reversal of TME immunosuppression. Whether peripheral immune changes translate to intratumoral immune reactivation remains unclear and represents a critical gap in the evidence base.

A particularly instructive set of negative findings deserves explicit discussion. The sertraline trial in patients with advanced cancer was terminated early because of a potential survival disadvantage (23), and subsequent meta-analyses in glioma have found no significant association between antidepressant use and improved survival (24). These null results suggest at least three interpretations: (a) the effects of antidepressants on survival may not be a class effect and may be drug-specific; (b) depressive symptom improvement alone may be insufficient to reverse an already established trajectory of tumor progression; and (c) single-node interventions (SSRI alone or β-blocker alone) may be inadequate to disrupt the coupled dysregulation across all three systems. The negative sertraline trial (23) may partly reflect its inclusion criteria-patients with major depression were excluded, potentially removing the subgroup most likely to derive immune-modulatory benefit from antidepressant therapy. While these interpretations remain *post hoc*, they provide indirect support for the multi-node intervention strategy proposed in our framework and underscore the urgent need for well-designed RCTs rather than reliance on observational data.

## 3 Synthesis, limitations, and a forward roadmap

### 3.1 A networked, systems-level, not linear model

The three systems described above are not independent. More fundamentally, they represent the interface of three interacting master regulatory systems: the autonomic nervous system (sympathetic driver), the neuroendocrine system (HPA amplifier), and the neuroimmune system (effector hub). HPA activation promotes immune suppression and BDNF downregulation. Sympathetic overdrive simultaneously fuels tumor cell-intrinsic aggressiveness and suppresses antitumor immunity, while also sensitizing the HPA axis to further activation. Inflammatory cytokines from the TME feed back to the brain to perpetuate both sympathetic and HPA hyperactivity. BDNF dysfunction may impair neuroendocrine feedback and modulate autonomic outflow at hypothalamic centers. We propose that the convergence of autonomic, neuroendocrine, and neuroimmune dysregulation creates a self-reinforcing, systems-level hub that may drive tumor progression. No single system is primary; each can amplify or buffer the others through positive and negative feedback loops. This networked property distinguishes our framework from linear causal chains and suggests that multi-node interventions (e.g., combining an antidepressant with a β-blocker and an exercise program) may be more effective than targeting any single pathway alone. The shift from “pathway thinking” to “systems thinking” carries immediate clinical implications: restoring homeostasis across autonomic, neuroendocrine, and immunedomains — not simply blocking a single cytokine or elevating BDNF — may be required for meaningful survival benefit. This systems-level model is presented as a working hypothesis to organize existing evidence and guide future research.

### 3.2 Critical limitations

We explicitly acknowledge three major limitations that preclude causal inferences.

#### 3.2.1 Heterogeneity in biomarkers and assessment tools

Differences in BDNF assay methods (serum vs. plasma, ELISA platforms), cytokine panels (varying combinations of IL-6, TNF, CRP), cortisol sampling protocols (single time point vs. diurnal rhythm), autonomic measures (heart rate variability vs. plasma/urinary norepinephrine vs. no assessment), and depression instruments (PHQ-9, Hospital Anxiety and Depression Scale (HADS), Composite International Diagnostic Interview (CIDI)) prevent the quantitative synthesis and meta-analysis of the proposed systems-level pathways. The heterogeneity in cytokine panels across studies remains a major barrier to quantitative synthesis. Although McFarland et al. (32) demonstrated consistent associations for IL-6, TNF, and CRP across 54 studies, the number of studies for other markers (e.g., IL-5, IL-13, albumin, NLR) was too small to draw firm conclusions, highlighting the need for standardized biomarker panels in future research.

Unresolved causality: The association between depression and reduced cancer survival could be explained by reverse causality (cancer progression causing depressive symptoms), shared etiological factors (e.g., chronic inflammation driving both), or residual confounding (e.g., smoking, alcohol consumption, and poor nutrition). Furthermore, the direction of causality is complicated by the fact that cancer-related inflammation and treatment-related adverse effects (e.g., chemotherapy-induced cognitive impairment, radiation fatigue) can independently trigger or exacerbate depressive symptoms. This reverse pathway from cancer to depression must be accounted for in any causal model. Our framework is therefore explicitly bidirectional, acknowledging that the neuroimmune dysregulation in cancer can be a primary driver of mood pathology, which may in turn accelerate disease progression. These caveats underscore that the proposed framework, while mechanistically plausible, requires prospective validation before causal claims can be made.

#### 3.2.2 Preclinical-to-clinical gap

Antitumor effects of antidepressants and β-blockers are primarily derived from animal models; human RCTs with survival endpoints are lacking.

Taken together, these caveats underscore that the tripartite framework proposed here is intended as an integrative working model to organize existing evidence and guide hypothesis-driven research, rather than a settled causal conclusion. Definitive causal relationships between depression and cancer survival have not been established, and all therapeutic strategies discussed remain investigational until validated in large-scale randomized controlled trials with survival endpoints.

#### 3.2.3 Evidence level

Meta-analysis > Observational > Preclinical. Cut-off values are exploratory and derived by the authors based on the distributions reported in the cited meta-analyses; they are intended for trial stratification only and do not represent clinically established diagnostic thresholds. All cut-offs require prospective validation before clinical application.

These candidate biomarkers correspond to the three endophenotypes proposed in Section 3.3.1. The HPA-dominant subtype would be defined by elevated cortisol and reduced BDNF; the inflammation-dominant subtype by elevated IL-6 or TNF; and the cytotoxicity-dominant subtype by reduced CD8^+^ proportion or low tumor IRF1 expression. Mixed phenotypes — meeting criteria for two or more subtypes — should be analyzed as a separate stratum in future trials. We emphasize that these cut-off values are derived from published literature and are intended for exploratory stratification only, not for clinical decision-making, until prospectively validated.

### 3.3 A testable future research agenda

Instead of simply advocating for “more research,” we propose a detailed hypothesis-driven roadmap based on the tripartite systems framework (**Table 1**). These hypotheses are not mutually exclusive but address complementary questions across the mechanistic and translational spectrum.

**Table 1:** Testable hypotheses generated by the tripartite systems framework.

Hypothesis	Approach	Timeline	Testability
H1: Critical time window	Prospective cohort comparing early (0–3 mo post-diagnosis) vs. delayed intervention	3–5 years	Feasible with serial biospecimen collection
H2: Causal direction	Mendelian randomization using genetic instruments for depression liability	1–2 years	High (publicly available GWAS data)
H3: Endophenotype- guided RCT	Biomarker-stratified trial (HPA/inflammation/cytotoxicity subtypes)	5–7 years	Requires prior validation of proposed cut-offs
H4: ICI combination	Phase II basket trial: ICI + SSRI + β-blocker vs. ICI + placebo	3–5 years	Testable only after phase I safety data

The first two hypotheses focus on foundational questions of timing and causality. H1 posits that the first 3 months post-diagnosisrepresenta critical window during which multisystem dysregulation is most pronounced and potentially reversible, which has direct implications for when to deploy biomarker monitoring or early intervention. H2 addresses the long-standing confounding and reverse-causality concerns in depression–cancer research; Mendelian randomization offers a way to test whether genetically predicted depression is causally linked to cancer outcomes without the biases that have plagued observational studies.

The latter two hypotheses translate the framework into clinical trial design. H3 moves beyond treating depression as a unitary condition by proposing biomarker-stratified trials — assigning patients to interventions based on their dominant endophenotype (HPA-axis, inflammatory, or cytotoxic). H4 builds on this logic to target a “high-risk, high-benefit” population, hypothesizing that patients with elevated inflammation and autonomic dysfunction may require co-administration of SSRIs and β-blockers to overcome stress-induced resistance to immune checkpoint inhibitors.

In terms of prioritization, H2 is the most immediately feasible given publicly available GWAS data; H3 carries the highest long-term translational impact but requires prospective validation of the proposed cut-offs. H1 and H4 occupy intermediate positions, with H4 contingent on phase I safety data. This roadmap is intended as a flexible framework for iterative hypothesis-testing as evidence accumulates.

#### 3.3.1 Endophenotype-driven clinical trials

One reason that prior antidepressant trials in cancer patients have yielded inconsistent results may be that “depression” is treated as a unitary condition. We propose that distinct systems-level endophenotypes exist, each requiring a different therapeutic emphasis. Based on our tripartite systems framework, we propose three candidate subtypes (**Figure 2**). These subtypes are defined not by depression severity alone but by the dominant pattern of autonomic, neuroendocrine, and immune dysfunction. Future RCTs should stratify randomization based on these endophenotypes, not on depression diagnosis alone.

**Figure 2:**
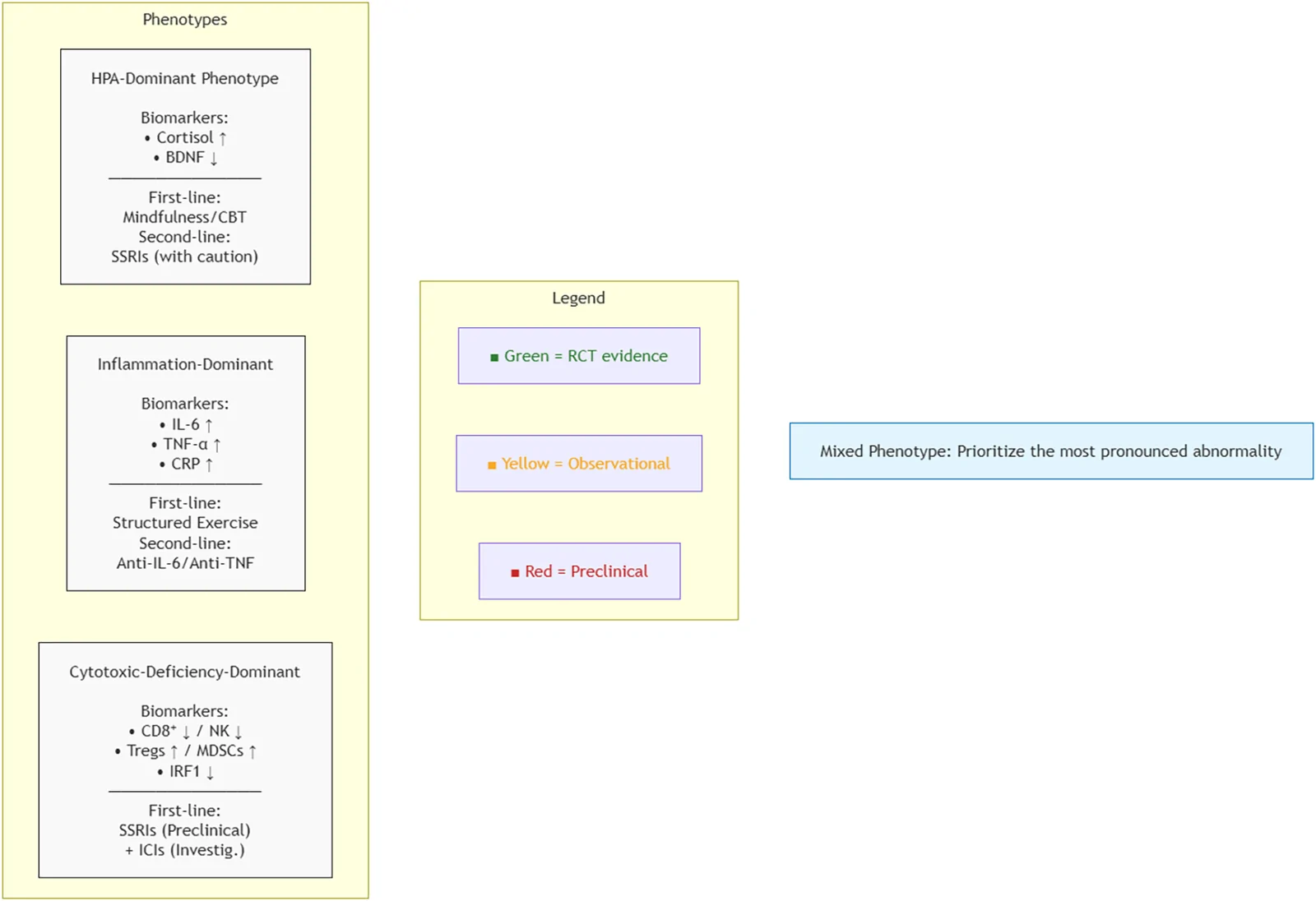
Tripartite neuroimmune endophenotypes and stratified intervention strategies for depressed patients with cancer. This image summarizes the three candidate endophenotypes proposed in Section 3.3.1. Based on the systems-level framework (**Figure 1**), these endophenotypes are proposed to guide biomarker-stratified interventions. The HPA-dominant subtype is characterized by elevated cortisol and reduced BDNF, with stress-management programs (mindfulness/CBT) as first-line and SSRIs (e.g., fluoxetine) with close monitoring as second-line. The inflammation-dominant subtype is defined by elevated IL-6, TNF, and CRP, with structured exercise as first-line and anti-inflammatory agents (investigational) as second-line. The cytotoxic-deficiency-dominant subtype features decreased CD8^+^/NK, increased Tregs/MDSCs, and reduced IRF1, for which SSRIs (preclinical evidence) combined with immune checkpoint inhibitors are proposed but remain entirely investigational. Importantly, the cut-off values for these subtypes are derived from published literature but are exploratory in nature; they are intended for trial stratification purposes only and do not represent clinically established diagnostic thresholds (see **Table 2** for details). Notes: Endophenotypes are not mutually exclusive; mixed presentations are common. All survival-related recommendations are hypothesis-generating; no intervention has demonstrated survival benefit in Phase III randomized controlled trials. Intervention colors indicate evidence level: green (RCT-supported for symptom improvement), yellow (observational/meta-analysis support), and red (preclinical/investigational only).

To operationalize these endophenotypes for future trial design, we propose the following exploratory cut-off values (summarized in **Table 2**), based on published literature and requiring prospective validation: HPA-dominant subtype defined as plasma cortisol > 1.5× upper normal limit AND BDNF below the lower normal limit; Inflammation-dominant subtype defined as IL-6 > 5pg/mL OR TNF > 8 pg/mL (above the 75th percentile of the general population); Cytotoxic-deficiency-dominant subtype defined as CD8^+^T cell proportion < 15% of peripheral blood lymphocytes OR IRF1 expression below the tumor tissue median. For patients meeting criteria for two or more subtypes, a ‘mixed phenotype’ category should be assigned and used as an independent stratification factor in clinical trials. We emphasize that these cut-offs are exploratory and must be validated in prospective cohorts before being adopted for clinical decision-making.

**Table 2:** Key biomarkers for operationalizing neuroimmune endophenotypes in future trials.

Biomarker biological domain		Proposed evidence level cut-off exploratory	
Plasma cortisol HPA axis		>1.5×upper normal limit	Observational (16, 30)
Serum BDNF	Neurotrophic	Below lower normal limit	Observational (18, 19)
IL-6	Systemic inflammation	>5 pg/mL	Meta-analysis (32)
TNF-α	Systemic inflammation	>8pg/mL (above75th percentile)	Meta-analysis (32)
High-frequency HRV	Vagal tone	< age-adjusted 5th percentile	Observational (41)
Peripheral CD8^+^ T cell %	Cytotoxic immunity	Reduced relative proportion	Preclinical/observational (64, 71)
Tumor-IRF1 expression	Immunometabolic	Below tissue median	Preclinical (20)

#### 3.3.2 The critical time window hypothesis

We hypothesize that the first 3 months after cancer diagnosisrepresenta critical window during which multisystem dysregulation is most pronounced and the pathological loop remains reversible. The negative results of the mirtazapine trial in established cachexia (48) underscore a critical principle: intervening after cachexia is fully manifested may be too late. Although mirtazapine did not improve appetite in patients with refractory cachexia, this does not rule out efficacy in pre-cachectic patients with concurrent depressive symptoms. Early depression intervention (within this window) may yield larger survival benefits than delayed or reactive treatment. This hypothesis requires prospective studies comparing early vs. delayed intervention, with serial multisystem biomarker assessments (cortisol, norepinephrine, heart rate variability, BDNF, cytokines, and immune cell subsets).

#### 3.3.3 Causal inference using mendelian randomization

To address the causality question, we recommend Mendelian randomization studies that use genetic variants associated with depression liability as instrumental variables to test whether genetically predicted depression is causally associated with specific cancer outcomes. This approach is not subject to reverse causality or many confounding factors.

#### 3.3.4 The “high-risk, high-benefit” immunotherapy hypothesis

Building upon the endophenotype framework above, we propose a dedicated phase II/III trial design in which patients with advanced cancer and moderate-severe depression (Patient Health Questionnaire-9 (PHQ-9)≥10) are randomized to ICI +SSRI + β-blockervs. ICI + placebo, and stratified by baseline inflammatory and autonomic phenotype. Primary endpoint: progression-free survival; secondary endpoints: overall survival and change in TME immune infiltrates (paired biopsies if feasible) and autonomic function metrics.

This triple combination (ICI +SSRI + β-blocker) is currently a purely investigational hypothesis, extrapolated from limited preclinical evidence. Before any clinical recommendation can be made, phase I/II trials are urgently needed to establish safety and tolerability, particularly given the theoretical risk that SSRIs may enhance immune-related adverse events when combined with ICIs, and that β-blockers may mask early warning signs of hemodynamic compromise. We strongly recommend that this hypothesis be tested only within rigorous research ethics frameworks.

### 3.4 Clinical practice recommendations

Given the current evidence level, we offer provisional recommendations that should be updated as RCT data emerge. These recommendations are graded by evidence level using the following convention, which corresponds to the color coding in **Figure 2**: Grade A(green) — supported by RCT evidence for symptom improvement (but no survival benefit demonstrated); Grade B(yellow) — supported by observational studies or meta-analyses; Grade C(red) — preclinical or theoretical (investigational).

#### 3.4.1 Screening

Two-step approach (Patient Health Questionnaire-2 (PHQ-2) → PHQ-9) for all newly diagnosed cancer patients, ideally within the first 3 months. Consideration should be given to adding simple autonomic metrics (e.g., resting heart rate, orthostatic response) as exploratory indicators (Grade B).

#### 3.4.2 Mild depression

Non-pharmacological interventions (exercise, mindfulness) as first-line (Grade A for symptom improvement). These interventions have documented effects on sympathetic tone and heart rate variability (Grade B).

#### 3.4.3 Moderate-severe depression

Pharmacological treatment (SSRI) combined with non-pharmacological interventions (Grade A for symptom improvement; survival benefit investigational). For patients with overt signs of sympathetic overdrive (tachycardia, hypertension, elevated norepinephrine), adjunctive β-blockers warrant investigation in clinical trials, though they are not yet standard of care (Grade C, investigational only).

#### 3.4.4 Referral

Psychiatry/psycho-oncology involvement for refractory or complex cases (Grade B).

We emphasize that these recommendations are based on evidence for symptom improvement and quality of life, not yet on survival benefit. The systems-level neuroimmune framework provides biological plausibility, but definitive clinical recommendations await large-scale RCTs with survival endpoints.

## 4 Discussion

Depression is robustly associated with reduced cancer survival, and this association is biologically anchored in the coupled dysregulation of autonomic, neuroendocrine, and neuroimmune networks. Rather than operating as independent pathways, these systems form a self-reinforcing hub in which sympathetic overdrive, HPA hyperactivity, and peripheral inflammation perpetuate one another, collectively remodeling the TME toward immune evasion and accelerated progression. This systems-level framework offers a biologically plausible explanation for the limited success of single-target interventions and provides a rationale for biomarker-stratified multi-node therapeutic strategies. Translating this framework into clinical benefits will require prospective trials that treat depression not merely as a psychiatric comorbidity but as a modifiable, systems-level determinant of cancer trajectory while maintaining scientific caution until survival endpoints are validated.
